# CCA: Cost-Capacity-Aware Caching for In-Memory Data Analytics Frameworks

**DOI:** 10.3390/s21072321

**Published:** 2021-03-26

**Authors:** Seongsoo Park, Minseop Jeong, Hwansoo Han

**Affiliations:** 1 Department of Electrical and Computer Engineering, Sungkyunkwan University, 2066 Seobu-ro, Jangan-gu, Suwon 16419, Korea; ss.park@skku.edu; 2 Department of Computer Science and Engineering, Sungkyunkwan University, 2066 Seobu-ro, Jangan-gu, Suwon 16419, Korea; ms.jeong@skku.edu

**Keywords:** big data analytics frameworks, caching optimization, in-memory data

## Abstract

To process data from IoTs and wearable devices, analysis tasks are often offloaded to the cloud. As the amount of sensing data ever increases, optimizing the data analytics frameworks is critical to the performance of processing sensed data. A key approach to speed up the performance of data analytics frameworks in the cloud is caching intermediate data, which is used repeatedly in iterative computations. Existing analytics engines implement caching with various approaches. Some use run-time mechanisms with dynamic profiling and others rely on programmers to decide data to cache. Even though caching discipline has been investigated long enough in computer system research, recent data analytics frameworks still leave a room to optimize. As sophisticated caching should consider complex execution contexts such as cache capacity, size of data to cache, victims to evict, etc., no general solution often exists for data analytics frameworks. In this paper, we propose an application-specific cost-capacity-aware caching scheme for in-memory data analytics frameworks. We use a cost model, built from multiple representative inputs, and an execution flow analysis, extracted from DAG schedule, to select primary candidates to cache among intermediate data. After the caching candidate is determined, the optimal caching is automatically selected during execution even if the programmers no longer manually determine the caching for the intermediate data. We implemented our scheme in Apache Spark and experimentally evaluated our scheme on HiBench benchmarks. Compared to the caching decisions in the original benchmarks, our scheme increases the performance by 27% on sufficient cache memory and by 11% on insufficient cache memory, respectively.

## 1. Introduction

A huge amount of sensed data is generated in real-time from IoTs and wearable devices and transmitted to the cloud for processing. As those devices are manufactured in lightweight forms, computing on large-scale data directly on them is often impractical. To this end, edge computing frequently utilizes serverless computing on the cloud, which is gaining popularity in cloud computing paradigms. The data collected from IoTs and wearables vary in their types and formats. Thus, additional data processing is required such as integration and classification [1]. Distributed data analytics frameworks are widely adopted for such data processing in the cloud. As processing large-scale data is a major challenge to big data analysis, many data analytics frameworks have emerged rapidly. Recently, in-memory data analytics frameworks have received the spotlight [2,3,4,5]. Providing machine learning libraries, these frameworks are adopted for application acceleration in the field of machine learning. They store intermediate results in memory and use them in iterative tasks, which greatly improves the performance over traditional storage-based processing frameworks. Apache Spark, currently the most popular in-memory data analytics framework, provides cache-related APIs for programmers to determine data to cache manually. Spark programmers may be able to utilize the cache more effectively with better knowledge of application logic, but the cache optimization can be additional overheads for programmers who want to focus on application logic.

For in-memory data analytics frameworks, many policies on cache eviction have been investigated to overcome the shortcomings in traditional policies, such as LRU, LFU, and FIFO. However, the execution times are easily affected due to garbage collection (GC) overhead. Even with excellent eviction policies, limitations exist to achieve the optimal performance. In our study, we found caching the adequate amount of data is equally important to performance. As most data analytics frameworks operate with the directed acyclic graph (DAG), which is the lineage information of the data processing, deciding which data to cache from the execution flow specified in the DAG is an appropriate approach but still not trivial [6,7]. A naive approach is to cache all data that are used multiple times. However, caching without considering execution context on cache capacity may increase the cache usage, which may incur GC overhead due to excessive memory demand. In addition, naive approaches may evict the important data for later computations and incur heavy data recomputation. Sophisticated approaches take into account the caching benefits of individual data. The benefits can be estimated by analyzing the execution flow and the cost of recomputation. On existing data analytics frameworks, the cost metrics for individual operators are rarely provided. Thus, programmers often find difficulties to figure out the benefits and cost overheads at the application development stage. Moreover, the caching decision from programmers may not be optimal. As the execution contexts on available cache amount is dynamically determined at run time, programmers cannot incorporate the run-time knowledge into statically written programs. When the cache memory is insufficient to store additional data, existing blocks are evicted from the cache. When the evicted blocks are referenced again, they are recomputed and re-stored in the cache. If this situation occurs repeatedly, it would be better to keep the data which require heavier recomputation cost than the other data. Meanwhile, when the cache capacity is sufficient to hold both, keeping both would result in the best performance. A proper caching decision for an execution context cannot guarantee optimal performance for the other execution contexts. Thus, accurately analyzing the execution flows and adapting to various execution contexts is necessary to achieve optimal caching decisions and this is not suitable for programmers.

In this paper, we propose a cost-capacity-aware caching (CCA) for in-memory data analytics frameworks. Our approach can be applied to the frameworks where the execution of jobs is described in a DAG. CCA makes caching decisions to maximize the predicted performance benefit based on the computing cost model. To this end, we established the operator-level metrics to represent the cost of individual operators in the distributed environment and built a computing cost model that predicts the cost of operators in terms of the size of input data. In addition to computing cost, caching decision in CCA depends on execution contexts, such as cache capacity, input data size, and execution stage progress. Using execution contexts, CCA dynamically updates the caching decision each time before running a job. Unnecessary data in the cache after finishing a job are continuously marked and those spaces are used for caching new data for the next jobs.

We evaluate the performance of CCA with Spark benchmarks from HiBench. According to our experiments, CCA never aggravates the performance of original caching decision in the benchmark programs, but finds the opportunities to improve the performance significantly in nine benchmarks out of 14 benchmarks. Compared to the original caching decision in benchmarks, our experimental evaluation shows that CCA achieves 27% speedup for application performance, when the cache memory is sufficient. On insufficient cache memory, CCA achieves 11% performance gains, where dynamic caching decision per job should be much more effective to draw the performance improvement. The rest of the paper is organized as follows. Section 2 provides background and motivation for our work. Section 3 describes the architecture and algorithm of CCA. Section 4 and Section 5 present our evaluation methodology and results. Section 6 discusses the related works, and Section 7 concludes our work.

## 2. Motivation

In this section, we analyze the application source code of a representative in-memory data analytics framework and explain the execution flow on the analytics engine described in DAG. Then, we discuss the limitation of fine-grained computing time metrics of the existing analytics frameworks. Finally, we present a motivating example to understand the impact of memory pressure.

### 2.1. Application Code Analysis of In-Memory Data Analytics Framework

**Figure 1:**
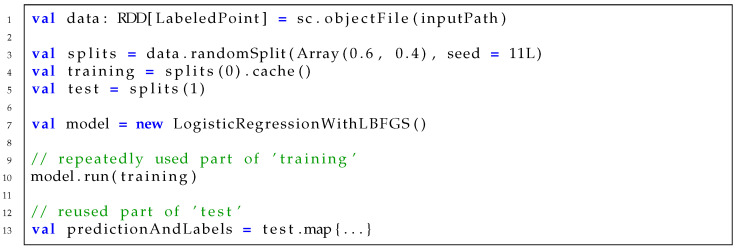
Source code of logistic regression on Spark.

Figure 1 shows the partial source code of logistic regression on Spark. As the intermediate data, training, are repeatedly used inside the model run in line 10, caching split(0) and copying its reference to training in line 4 will result in performance benefit. Another intermediate data, data, which are created in line 1, are reused to generate splits in line 3. These data could have been selected to cache, but not cached. Thus, when splits(1) is copied to test in line 5 and used in line 13, all the operators in its RDD lineage should be executed. This includes a repeated file read of an input file in inputPath. Caching both data and training can avoid repeated file reads, but it may cause performance degradation due to insufficient memory. The original benchmark makes an empirical decision to cache training, as it is repeatedly used in the model run. However, it is not always optimal under different execution contexts.

### 2.2. Execution Flow and Cached Dataset

Execution flow of data analytics frameworks such as Spark [2], Hadoop [8], and Dryad [9] can be represented by a directed acyclic graph (DAG)  [10]. Each edge represents dependencies between intermediate results in the execution flow. Each node of DAG represents the dataset generated by the operator. An operator transforms the input dataset into an output dataset. The whole execution flow of an application consists of multiple jobs. A job is composed of stages. A stage has a boundary around operators that have wide dependencies. DAGs in the analytics framework can have two types of data dependencies: narrow dependencies and wide dependencies. In narrow dependencies, each block of dataset depends on one block of the previous dataset. Meanwhile, wide dependencies appear when each block of the dataset may be dependent on multiple blocks of the previous dataset. A stage is executed on a set of tasks and each task takes blocks of the dataset. In data analytics frameworks, the first dataset usually consists of data blocks generated by raw input data from the storage system (e.g., HDFS [11], Amazon S3 [12], and Tachyon [13]), and the number of tasks is determined by the amount of data blocks. The final output of the stage is delivered to the input of the next stage.

**Figure 2:**
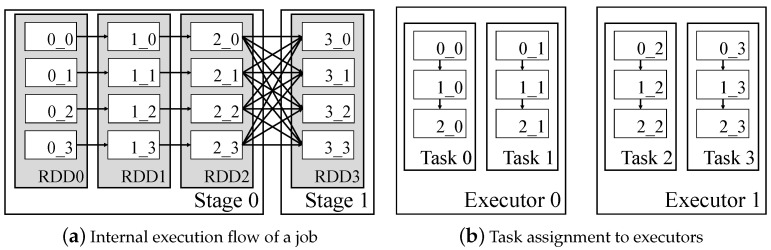
Internal representation of execution flow and task execution for blocks in Spark: *x_y* in blocks denotes dataset id (*x*) and partition id (*y*).

Figure 2a shows an internal representation of execution flow in Spark. The operator is invoked on a resilient distributed dataset (RDD), which is an abstraction of a dataset [14]. In the example in Figure 2a, each RDD is partitioned to four blocks, and a new block is created as a result of operator execution. Figure 2b shows a task assignment in executors. Among the assigned tasks in executors, runnable tasks are executed in parallel. As a dataset in a stage is composed of four blocks in the example, four tasks are created and evenly assigned to available executors. Execution plan of a task is internally represented as [0_y→1_y→2_y]. If RDD1 in the example is cached, four blocks from RDD1 (consisting of 1_0,1_1,1_2, and 1_3 blocks) will be stored in the Spark cache memory. When a task is ready to execute, its DAG is traversed with depth-first search, and operators are started from the deepest cached node. Assuming RDD1 is cached in the example, the operators to generate the blocks of RDD2 from the blocks of RDD1 will start to execute in all tasks. As such, it is possible to distinguish intermediate data that is highly reusable through the DAG information of the application, and it can be selected as an appropriate caching candidate.

In many applications, some datasets from operators are reused on iterative jobs. To improve the performance of the application, caching for iterative jobs is beneficial. If datasets generated by reused operators are not cached, recomputation of the iterative operators is required, which degrades the performance. Most existing frameworks do not provide sophisticated caching for repeatedly performed operators. Although Spark supports APIs to cache the dataset specified by the programmer, it rather causes development overhead. Our goal is to provide a caching mechanism in consideration of execution flow and potential performance increase for repetitive jobs.

### 2.3. Task-Level vs. Operator-Level Timing Metrics

It was confirmed that intermediate data to be reused can be selected through the above source code analysis (Section 2.1) or execution flow analysis (Section 2.2). In addition to data reuse times, for optimal performance, it is necessary to compare execution cost through recomputing for recovery and performance gain due to caching. There is a study verified that the execution cost of each operator can be an important feature for cache memory management, through a cost-aware eviction policy to replace LRU which is the default of Spark [15,16]. In order to measure the recomputing time of intermediate data, the cost of the ancestor operators of the data must be measured. Unfortunately, existing data analytics frameworks only provide the task-level computing time metrics [2,8]. The executor only records times when a task starts and finishes. Inferring the performance benefit of caching has two limitations with the existing task-level metrics: First, task-level timing is a local metric measured on an individual executor. Blocks in a dataset are distributed across multiple tasks and processed on multiple executors in a distributed environment. Second, the task-level timing is too coarse to calculate the performance benefits from operator-level computing. To estimate the benefit of caching, skipped block processing times in a task should be measured. In our work, new operator-level timings are integrated over the distributed environment and proportionally matched with stage timings to build a computing cost model.

### 2.4. Memory Pressure and Performance

In-memory analytics frameworks store intermediate data in the cache memory to improve the performance of iterative operations. If the size of the dataset to cache is larger than the capacity of the cache memory, the performance of the framework may degrade [17]. RDDs used with cache() are stored in the cache memory when the corresponding operators are processed. They evict data according to LRU policy if they cannot keep all datasets to the cache. When evicted RDDs are referenced again, they are recomputed and stored in the cache memory. As recomputed RDDs move back to the cache memory, other existing RDDs in the cache memory can be evicted again. The process of recomputation and eviction due to the lack of cache memory degrades the performance of frameworks. To alleviate the situation, adjustment to the current caching decision is necessary, if the cache memory cannot keep all the cached data.

**Figure 3:**
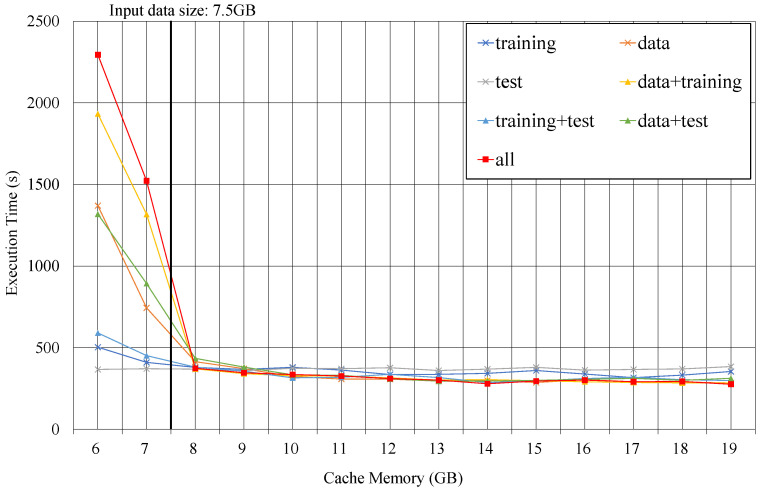
Execution time of logistic regression on 7 different caching decisions.

Figure 3 plots the execution times on various capacities of the cache memory for logistic regression on Spark. Each line represents the caching decision for the dataset in example code of Figure 1. It shows all seven combinations that can be cached for the three intermediate data: data, training, and test. When the cache memory is smaller than input data size (7.5 GB), the execution times for two caching decisions—all and data+training—take much longer than the other caching decisions. As the lack of cache memory causes the recomputation of the data block, the performance degrades for caching decisions to hold larger data than the capacity. In the case of caching test only, the number of reuses is very small, so performance tends to be the worst when cache capacity is sufficient. On the contrary, in a situation where the cache capacity is extremely small, the caching test reduces the burden on the cache and shows the fastest performance. When the capacity of cache memory is sufficient more than 15 GB, caching all achieves the best performance. From this experiment, we verify that one caching decision cannot be optimal for all execution contexts. Thus, the caching decisions should be changed depending on the execution context to achieve best performance.

## 3. Design Decisions

In this section, we present the system design of CCA, which makes the caching decision based on the computing cost model in consideration of execution contexts. We describe our elaborate implementation in Spark. Additionally, we describe detailed methodologies to make the caching decision through two algorithms. Table 1 is the glossary of notations used in this paper.

### 3.1. Cost Model and Caching Benefit

In the previous section, we noted the limitation of existing task-level computing time metrics to build the computing cost model [18]. Existing frameworks do not measure the individual block computing time and local task-level metrics cannot represent the computing time from the perspective of the distributed environment. We establish an operator-level metric by integrating operator times on all tasks to determine the computing cost of the operator in the execution flow. We split the task into individual block computing and measure the computing time of each block. Initially, we tried to estimate the operator computing cost as the maximum value of the computing time of blocks in the dataset. However, one major challenge we faced with the initial estimation is that multiple tasks can be assigned per executor core. The executor can perform tasks in parallel as many as the number of cores. If the number of tasks is greater than the number of available cores, the number of tasks processed by one core can be multiple. In this case, the cost of computing the dataset by the operator cannot be determined by the maximum block computing time. Our approach to addressing this challenge is matching the sum of the dataset’s block computing time proportionally to the stage duration. Assuming that the stage contains *n* tasks. In Equation (1), $T_{i}$ is the total computing time of blocks generated by $O_{i}$, where $T_{i_{j}}$ is the computing time of $D_{i_{j}}$.

(1) $$T_{i}=\sum_{j=1}^{n}T_{i_{j}}$$

In frameworks that adopt BSP model [19], such as Spark or Hadoop, a stage finishes only when the last task is completed. Stage duration can be obtained as the time from the start of the first task to the end of the last task. In Equation (2), $C_{i}$, the estimated computing cost of $O_{i}$, is defined by matching the ratio of $T_{i}$ to the sum of the $T_{x}$ of all *m* operators in the stage to the stage execution time *S*. For the computing costs of reused operators at multiple stages, the averages of the measured computing costs are used.

(2) $$C_{i}=\frac{T_{i}}{\sum_{x=1}^{m}T_{x}}\times S\;$$

Measured computing cost by operator-level metric depends on the size of the input file and is not generally applicable to the different size input files. We build a computing cost model based on the measured operator-level metrics in terms of the input file sizes. We measure the operator-level metrics for three representative sizes of input data and calculate the linear trend model by using three computing costs from different input sizes. Our cost model predicts the costs of operators on a given size of input data through the linear trend cost model.

Based on the computing cost model, we define caching benefit as the reduced execution time which is decreased by caching the dataset. The caching benefit changes as iteration is performed, so the benefit must be recalculated for each job. The *a* stands for the nearest cached ancestor in DAG. The $I_{i}$ is a number of iterations for $O_{i}$. The benefit from caching the dataset generated by $O_{i}$ is calculated in Equation (3) as follows:

(3) $$B_{i}=\left(\sum_{x=i}^{a}C_{x}\times \left(I_{i}-1\right)\right)$$

Most applications that running in the distributed environment are recurring applications [20]. Our approach obtains the block computing time and the size of the dataset from the previous run.

To make a caching decision that maximizes caching benefit, all possible decision’s caching benefits should be compared. The number of possible caching decisions with *k* operators in the execution flow is $2^{k}$. As *k* increases, the cost of comparing all possible caching decisions increases exponentially. Even if the caching decision selected from our approach shows sub-optimal performance, the completeness of making a caching decision must be guaranteed. To address this problem, we propose a DAG clustering method, which clusters nodes with the same iteration count from the job DAG. Each node represents the dataset in the execution flow of an application. The iteration count of the dataset is defined as the number of job DAG that records the dataset. The operator that creates the dataset in the execution flow is used as much as the iteration count.

Considering the execution process of the analytics framework, only one dataset in the cluster needs to be cached. When two nodes in the job DAG are adjacent, the child node is always created from the parent node. If both nodes have the same iteration count, both nodes are referenced in the same job DAGs. Therefore, datasets in the cluster will be referenced in the same job DAGs. If the cluster contains cached nodes, only the child nodes of the cached nodes in the cluster need computation. Considering the characteristics of DAG, only the bottom of the nearest cached node is referenced, so only one node in the cluster needs to be cached. Caching can be specified based on the cluster in which the entire DAG is divided into subgroups. Thus, DAG clustering narrows down candidates for caching decisions and reduces the cost of selecting the caching decision. The dataset with the highest caching benefit in the cluster is selected as the dataset to be cached. The caching benefit of the cluster is defined as the caching benefit of the selected dataset.

**Figure 4:**
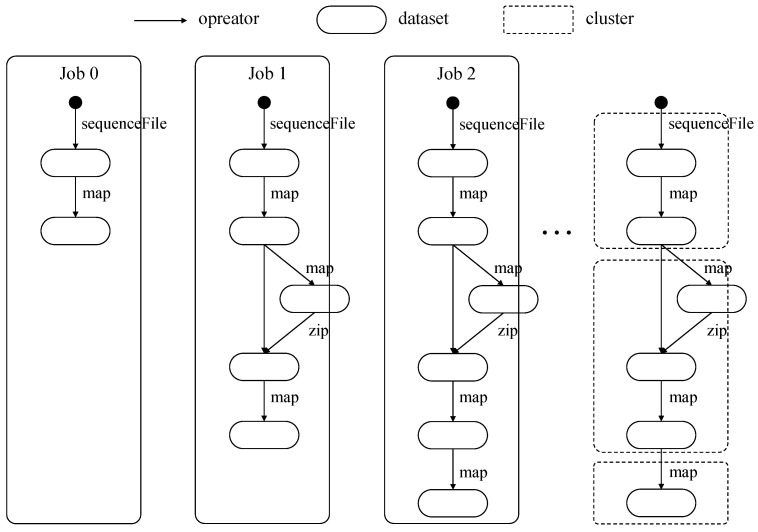
Part of KMeans’s job directed acyclic graphs (DAGs) and example of DAG clustering.

Figure 4 shows part of KMeans workload’s job DAGs. Our clustering method starts the clustering from DAG’s root node and nodes with the same iteration count are separated. Sequence [sequenceFile→map] is used from job 0 to job 2, and the dataset generated by sequence [sequenceFile→map] is referenced three times in the example. Sequence [map→zip→map] is used from job 1 to job 2, and the dataset generated by sequence [map→zip→map] is referenced two times in the example. Sequence [map] is only used in job 2. Datasets in all job DAGs are clustered into [sequenceFile→map], [map→zip→map], and [map] according to the number of using.

### 3.2. Spark Implementation

**Figure 5:**
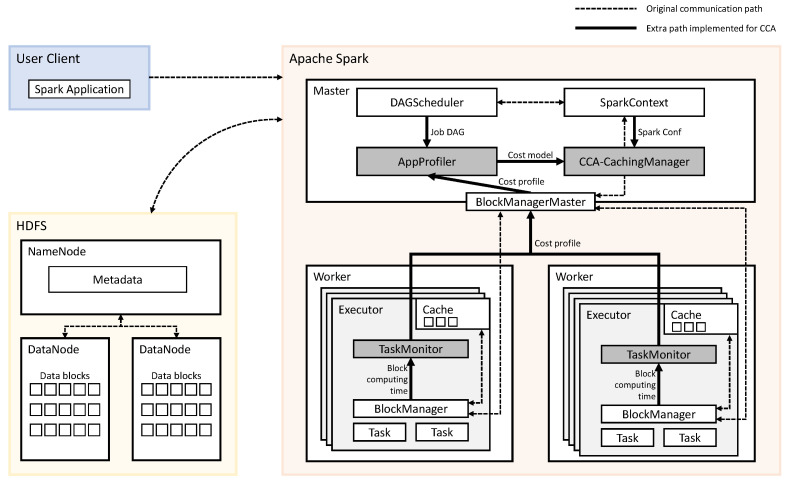
Architecture of cost-capacity-aware caching (CCA).

Figure 5 gives an overall architecture of CCA. We have implemented CCA in Spark, and shaded components are the main implementations in Figure 5. AppProfiler and CCA-CachingManager are implemented on a master node of distributed Spark. TaskMonitor is implemented on each worker node of distributed Spark. The other components, DAGScheduler, SparkContext, BlockManagerMaster, and BlockManager, are default components of Spark.

Before running the application for the first time, the AppProfiler collects the necessary information of application for building computing cost model. It collects DAGs, the iteration count of a dataset, the size of a dataset, and computing cost of blocks. Iteration count and DAG information are obtained from the DAGScheduler. Distributed TaskMonitor collects the computing time of data block for each task from the BlockManager and sends it to the BlockManagerMaster. The BlockManagerMaster uses the collected information to determine the computing costs of the operator and sends it to the AppProfiler. After profiling, the AppProfiler sends the cost model of an application to CCA-CachingManager.

The main algorithm to make a caching decision is implemented in CCA-CachingMan-ager. When an application is submitted through spark-submit scripts, Spark launches the driver with an object called SparkContext. SparkContext provides access to the various components on the distributed Spark. One component of the distributed Spark is SparkConf, which gives the information such as a number of executors and executor’s capacity of the memory. CCA-CachingManager makes a caching decision by using the profiled results received from AppProfiler and configuration information from SparkConf.

### 3.3. Caching Decision Algorithm

In the previous section, we proposed a clustering method for the caching decision. The pseudocode for the DAG clustering and caching decision in CCA is described in Algorithm 1 and Algorithm 2. Two algorithms are implemented in CCA-CachingManager component.

We formalize the procedure of clustering the DAG in Algorithm 1. As briefly described above, nodes with the same iteration count will be clustered. The clustering method recursively traverses nodes of the DAG starting from *root*. The *clusters* is a set of a cluster that partially grouped from the DAG. The *descs* is a queue in which nodes whose iteration count should be compared to the nodes stored in *cluster* are stored. Empty *descs* means that all nodes in the job DAG are clustered, meaning there are no more nodes to traverse. If *descs* is not empty, consider whether to include *desc* in *cluster*. A *iter* stores the iteration count of all nodes, and the cluster’s iteration count is the same as the node in the cluster. If the iteration count of *desc* and the iteration count of *cluster* are the same, add child nodes of *desc* to *descs* and include the *desc* in the *cluster*. If they are not the same, start the clustering recursively with a sub-graph where *desc* is the root node. Finally, clustering results from all job DAGs are integrated to obtain a cluster set of the entire execution flow. The procedure of clustering is performed once after the application is launched.

We describe the procedure of making a caching decision in Algorithm 2. The procedure of clustering nodes of the DAG and extracting the cluster set of the application is involved in making a cache decision before the start of the first job. The *benefit* stores caching benefits of all clusters. The *cluster.dataset*, the dataset to be cached in the cluster, is the dataset with the highest caching benefit in the cluster. The caching benefit of the cluster is defined as the caching benefit of *cluster.dataset*. Initially, the algorithm updates the *benefit* according to the cost of the model and the remaining iteration count of the dataset. All clusters are candidates for caching and a cluster to be included in the caching decision is determined in order of the cluster’s caching benefit. If there is enough space in memory to store the selected dataset, include the dataset in *caches*. If the new dataset is added to *caches*, the caching benefit of clusters is updated.

CCA updates the remaining iteration count of the dataset and performs the procedure of making a caching decision each time before every job starts. The dataset included in *caches* is stored in the cache when used for the first time in the job. The dataset not included in *caches* is removed from the cache. Caching decision is made on the master node of the distributed Spark at the job running time of worker nodes. Required decision time for the next job is overlapped at the running time of the previous job. The decision time for the first job is overlapped at the time after the application launches and before the first job is submitted.
**Algorithm 1** A recursive algorithm for DAG clustering    **Input   :***iter*—map that stores the iteration count of the corresponding dataset or cluster                  *root*—top node in DAG
**    Output: ***clusters*—a set of clustered nodes
1: **function** clustering
2:     ▹ Recursively traverse all nodes in DAG
3:     *cluster*
←{root}
4:     *descs*
←{root}
5:     **while**
*descs*
≠⌀
**do**
6:         *desc* = *descs*.pop
7:         **if**
*iter*[*desc*] == *iter*[*cluster*] **then**
8:               *descs*
←descs∪desc.children
9:               *cluster*
←cluster∪{desc}
10:         **else**
11:               *clusters*
←clusters∪{cluster}
12:               *clusters*
←clusters∪clustering(desc)
13:               *cluster*
←⌀
14:         **end if**
15:     **end while**
16:     **return**
*clusters*
17: **end function**
**Algorithm2** A baseline algorithm for making a caching decision.    **Input    :***M*—size of total cache memory                  *U*—size of used cache memory
                  *benefit*—map that stores caching benefit of the corresponding cluster
                  *clusters*—a set of clustered nodes
    **Output:** *caches*—a set of candidates to cache
1:**function** make_decision
2:       update(*benefit*))
3:       *caches*
←⌀
4:       **for all**
*cluster*
**in**
*clusters*
**do**5:              **if**
*U* ≤ *M*
**then**6:                    *caches*
←caches∪{cluster.dataset}7:                    update(*benefit*)
8:              **end if**
9:         **end for**
10:       **return**
*caches*
11: **end function**


## 4. Evaluation Methodology

In this section, we demonstrate a methodology for evaluating CCA with 14 workloads in the Intel HiBench [21,22] benchmark suite. Among the 17 Spark workloads in HiBench, 14 workloads which provide a reused operator are used for the experiment. We use machine learning [23], graph computation [24], and other workloads to measure the performance of CCA. We measure the prediction accuracy of the caching benefit based on the measured operator-level metric and the prediction accuracy of the computing cost model. Then, we evaluate the performance of CCA on sufficient cache memory and reduced cache memory. To verify the efficiency of our proposed CCA, we compared it with cost-aware-only and best-combination. Cost-capacity-aware (CCA) selects caching candidates based on heuristics through DAG clustering, and corrects the candidates according to cache usage during execution. Cost-aware-only does not consider cache capacity and statically selects a cluster predicted with optimal performance through DAG clustering as a caching candidate. Best-combination is the fastest case among the execution results according to the combination of caching or not for all datasets.

In addition, CCA is compared with other cache memory management techniques that have proven to be more efficient than LRU. We implemented and evaluated CCA in Apache Spark. Table 2 provides information about the distributed environment that CCA is tested. We used NVMe storage and high-bandwidth Ethernet to configure the system for modern distributed environments. Our distributed Spark consists of one master node and two worker nodes and contains a total of 10 executors and 50 cores. For other configurations [25], default parameters are used. Our evaluation is measured on Ubuntu 14.04, Spark 2.1.0 standalone mode, and HDFS with Hadoop 2.7.2.

## 5. Results

The main results of this study relate to two aspects: prediction accuracy and performance evaluation. In summary, the proposed CCA generally showed high prediction accuracy, and the performance was also close to optimal.

### 5.1. Prediction Accuracy of Caching Benefit

**Figure 6:**
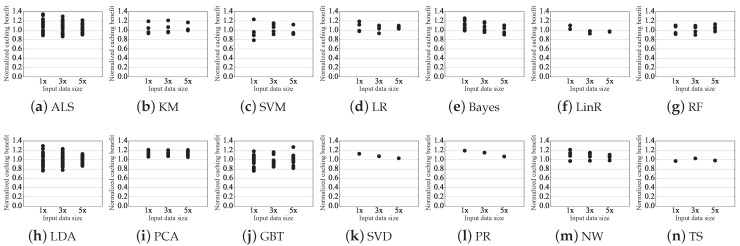
Predicted caching benefit normalized to actual caching benefit from the cluster measured on three sizes of input data.

We compare the predicted benefit with the actual benefit from caching the dataset in the reused cluster. The predicted caching benefit is calculated by Equation (3) based on the measured operator-level metric. The actual caching benefit is actual reduced execution time due to caching. In this evaluation, the operator-level metric is measured from three sizes of input data, 1x, 3x, and 5x. 1x, 3x, and 5x are 10%, 30%, and 50% of the size described in Table 3, respectively. The predicted caching benefit normalized to the actual caching benefit is used as the prediction accuracy for evaluation. Prediction accuracy for all reused clusters is plotted in Figure 6. In the case of SVD, PR, and TS, only one plot is plotted per input data because only one reused cluster exists in each workload’s DAG.

The predicted caching benefit for all reused clusters are within 26% of the actual caching benefit when measured in 5x, within 29% of the actual caching benefit when measured in 3x, and within 34% of the actual caching benefit when measured in 1x. The relative error tends to increase as the size of the input data decreases. The maximum error is relatively higher than the average error due to operators that take less time. On average, the predicted caching benefit of all reused cluster is 7% different from the actual caching benefit when measured in 5x, 9% different from the actual caching benefit when measured in 3x, and 12% different from the actual caching benefit when measured in 1x. For most operators used in ALS and GBT, the measured computing time is low: most have a cost of less than 1 s.

Exceptionally, the predicted caching benefit for LDA is within 13% of the actual caching benefit when measured in 5x, 23% of the actual caching benefit when measured in 3x, and 29% of the actual caching benefit when measured in 1x, despite the high operator computing time is measured. This is because the randomSplit() operator is mostly used for LDA, and deviation occurs whenever computing cost is measured.

### 5.2. Prediction Accuracy of Cost Model

**Figure 7:**
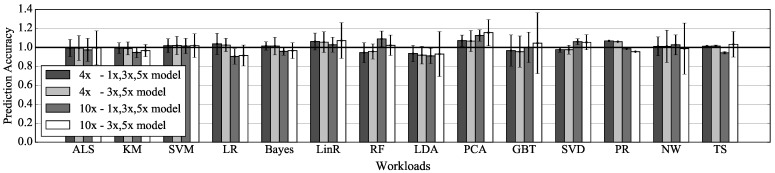
Prediction accuracy of two models according to the input data size for evaluation.

We compare the cost of the operator on the model with the cost measured by the operator-level metric. This comparison indicates how well the cost model predicts computing costs at various input data size. The cost on the model normalized to the cost measured by our metric is measured as the prediction accuracy of the model. In this experiment, the cost model trended with measured metric on 1x, 3x, and 5x is compared with the cost model trended with measured metric on 3x and 5x. The accuracy is measured within (interpolation) and beyond (extrapolation) the sequence of value used for trend line fitting. The input data 4x used for interpolation are 40% of the size described in Table 3, and the input data 10x used for extrapolation are the same as the size described in Table 3. The accuracy is measured for all reused operators, and the average and deviation of the accuracy for each model are shown in Figure 7.

Predictions of the cost model trended with three data are within 19% of the actual execution time when using interpolation, and within 23% of the actual execution time when using extrapolation. Predictions of the cost model trended with two data are within 19% of the actual execution time when using interpolation, and within 38% of the actual execution time when using extrapolation. In the case of interpolation, there is little difference between the prediction accuracy of the two models. However, in the case of extrapolation, predictions of the two models are noticeably different. It can be seen that the trend line equation obtained from the two data has limitations as the size of input data goes out from the range of the data. The accuracy of the cost model trended with two data indicates that the model could not be used to predict caching benefit for some workloads, such as GBT. Thus, CCA’s cost model is built using values measured from three sizes of data.

### 5.3. Performance on Sufficient Cache

**Figure 8:**
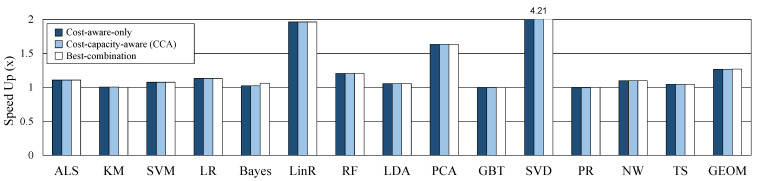
Comparison of three methods on sufficient cache memory.

In this section, we evaluate the performance of CCA and compare it with other caching methods. For the experiment, the capacity of the cache memory is configured sufficiently so that there is no dataset evicted from the cache. The maximum required cache size and provided cache size on sufficient cache memory for each workload are shown in Table 3. We run each workload 10 times and we average the results of each workload. Figure 8 shows the performance improvement of the three caching decisions compared to the default caching decision. CCA and cost-aware-only show the same performance because the caching candidate decision is the same when cache memory is sufficient. Best-combination has slightly more performance gains than the other two decisions. However, if the number of operators is *k*, best-combination can be obtained by running $2^{k}$ times. Actually, we had to spend a lot of time getting the best-combination, and this is distinctly limited to apply and use in real systems. The proposed CCA shows an average (geomean) of 27% speedup out of 14 workloads.

### 5.4. Performance on Reduced Cache

**Figure 9:**
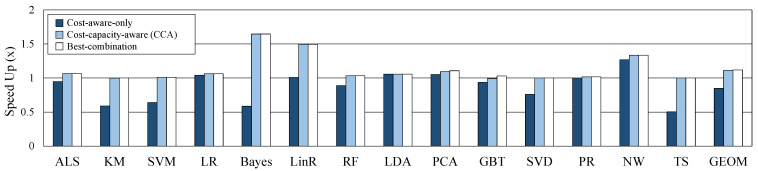
Comparison of three methods on reduced cache memory.

Assuming a system with insufficient memory, performance was compared by reducing the cache memory size. As shown in Table 3, we set about half of the max required cache size used in situations when memory is sufficient for each workload. The cache memory is managed by the Spark default LRU policy when memory is insufficient. Figure 9 shows the speedup of three methods to the performance of original caching on the reduced cache size. In case of cost-aware-only, a caching candidate is statically selected and execution context is not considered, so the max required cache size is the same as in the sufficient case. Therefore, the lack of cache capacity causes a lot of GC and performance degradation (geomean 15%). On the other hand, as CCA considers cache capacity as well as cost, it never exceeds the provided cache size, and performance does not worsen than the default in any case. CCA and best-combination improved geomean performance by 11.2% and 11.8%, respectively, and there is little difference although it takes a very large number of runs to obtain a best-combination. Through these experiments, the proposed CCA shown that operates efficiently in both cases of sufficient or insufficient memory. In addition, in big data processing, operator’s cost and cache capacity are important metrics to obtain optimal performance.

### 5.5. Comparison with LCS

**Figure 10:**
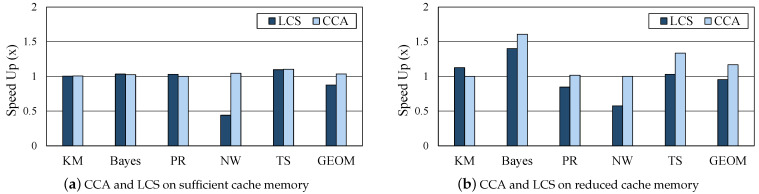
Comparison between the proposed CCA and other cache memory management techniques.

Least cost strategy (LCS) [16] is a cost-aware eviction policy for efficiently managing cache memory instead of the default LRU policy in the data analytics frameworks. CCA and LCS were compared with the environment shown in Table 2 and Table 3. LCS was able to successfully perform five out of 14 workloads: KM, Bayes, PR, NW, and TS. Figure 10 shows the performance of CCA and LCS, normalized to the original workload execution with LRU. Machine learning workloads such as KM and Bayes have frequent data reuses. For the two workloads, LCS showed a meaningful performance improvement when the cache memory is insufficient. As for NW, which is shuffle-heavy, there was a big performance degradation with LCS. In some workloads, the performance gain of LCS was slightly better than our proposed CCA, but CCA did not degrade for all workloads with sufficient or insufficient memory. As CCA is a method of finding the optimal caching decision per job, direct comparison to eviction policies may not be closely related. However, this experiment verified that the proposed CCA has an effect similar to adopting the efficient eviction policy, because the proposed CCA relieves the burden of GC through optimal caching selection even in the case of insufficient cache memory.

## 6. Related Work

**Analysis of Execution Flow.** Several studies improve the performance of iterative jobs through the analysis of execution flow. Meta-dataflows (MDFs) [26] illustrate a model for effectively performing an exploratory workflow in a distributed analytics engine such as Spark or Flink [27]. MDFs integrate iterative jobs to adjust the execution flow of the application. The modified execution flow omits redundant operations. MDFs include a cache replacement policy that takes into account reference count and data loading cost. Our work performs caching considering not only the branch of the integrated execution flow, but also the computing cost of all datasets.

S-CACHE [28] automatically makes a sub-optimal caching decision by analyzing the application’s execution flow and cost model, implemented in Apache Spark. It calculates the computational cost of individual caching decisions by considering the dataset’s computation cost, cache writes cost, and cache read cost. Then, it compares all possible caching decisions from execution flow statically. It selects the caching decision with the lowest cost. The performance of S-CACHE is evaluated on one specific application. It needs to be proved whether it is generally applicable to other applications. On the other hand, we build a computing cost model for various applications and evaluates the prediction accuracy of them. We make the distinction from S-CACHE by considering the execution context in addition to cost-aware caching.

LRC [15] and MRD [29] are proposed as the cache replacement policy of the analytics framework. These studies traverse the execution flow previously and decide replacement based on the collected information. LRC defined the reference count as the number of times to reference the data block in the execution flow of an application. Reference count is updated as the application runs. It is implemented so that data blocks with a low reference count are preferentially dropped from memory when the cache memory is insufficient. MRD defines the interval from the current execution point to the reference of the data block as a reference distance for the stage and job. It preferentially evicts data blocks with the highest reference distances. It includes the prefetching of the data to be referenced soon. No adjustment of execution flow for these studies is existing, therefore our research and these can be compatible.

**Memory Management.** There have been studies to enhance the utilization of cache memory of the analytics framework. Neutrino [30] and MemTune [31] dynamically adjust run-time parameters considering cache memory usage. Neutrino is a memory management system for the distributed framework, implemented in Spark. Spark provides several ways in which data blocks are stored, such as memory only, memory and disk, and no serialization. This study improves performance by adjusting the way of data blocks are stored at run-time. This is based on information from previous runs. Our work is orthogonal to this work in that it does not decide where to place the data block. Thus, this study can potentially be applied to our study.

MemTune dynamically manages a fraction of computation/cache memory to improve memory utilization for in-memory analytics frameworks. It monitors run-time statistics such as garbage collection time, task execution time, and size of the dataset. It analyzes the collected information and adjusts the cache size at run-time. It provides prefetching and eviction of a data block, using execution flow information from the DAG. This study seeks to overcome performance degradation due to memory pressure. Our study attempts to minimize performance degradation due to garbage collection and recomputation. However, this study focused on controlling memory contention, and it is different from our study to improve the performance of overall jobs.

**Comparison between CCA and other studies** In Table 4, the proposed CCA is compared with the existing related studies described in this section. Many other studies have argued that the execution flow should be considered in an in-memory big data processing framework where caching is important. In addition, it is rare that both the cost and memory capacity of each operator are considered. As we compared our CCA and LCS in Section 5.5, CCA directly and automatically determined the caching decision to optimize the execution flow and showed near-optimal performance no matter what the memory situation.

One study confirmed that existing typical cache allocation policies are not suitable in a cloud environment, and proposed fair and efficient cache sharing for big data analytics [32]. As such, many studies are underway to further optimize caching for big data analytics from various perspectives, and its importance is undeniable.

## 7. Conclusions

To process the sheer amount of sensing data generated from IoTs and wearables in real-time, improving the performance of distributed data analytics frameworks is a challenging area of research. This paper proposed CCA, a cost-capacity-aware caching optimization scheme. Our approach is generally applicable to DAG-represented in-memory data analytics frameworks. We devised an operator-level metric to obtain the computing costs of operators. The cost model was built based on the measured operation-level metric to predict the benefit from the caching dataset. Our scheme primarily selects dataset with the highest benefit from caching. CCA adjusts the caching decision considering the execution context, while the application is running. We implemented CCA on Apache Spark to evaluate the performance. For 14 workloads in HiBench benchmark, CCA achieved the performance gains of 27% on sufficient cache memory and 11% on insufficient cache memory, respectively. In addition, compared with other cache memory eviction policy (LCS), we found that CCA is effective in most situations. We have discovered that efficient caching is still important in in-memory analytics frameworks, even though it is configured with fast storage and network. In addition, it is burdensome for programmers to select data to cache manually, because the optimal performance can be obtained only if dynamic execution contexts are taken into account. It is often difficult to decide which data to cache beforehand without executing the application with actual input data. We believe our proposed CCA helps find the optimal performance, while reducing the efforts of application tuning in big data analytics frameworks.

## Figures and Tables

**Table 1:** Glossary of the notations.

Notation	Meaning
*n*	Number of blocks in the dataset
*m*	Number of operators used in the stage
*a*	Ancestor which is the nearest cached in DAG
*S*	Stage execution time
$O_{i}$	*i*th operator
$D_{i}$	Dataset generated by $O_{i}$
$D_{i_{j}}$	Data block of $D_{i}$
$T_{i_{j}}$	Computing time of $D_{i_{j}}$
$T_{i}$	Total computing time of blocks on dataset $D_{i}$
$C_{i}$	Estimated computing cost of $O_{i}$
$I_{i}$	Number of iterations for $O_{i}$
$B_{i}$	Benefit from caching $D_{i}$

**Table 2:** Distributed server hardware specification and Spark configuration.

Hardware (Node Specification)	
CPU	Intel Xeon E5-2640 v3 * 2
RAM	128 GB
Storage	1.5 TB NVMe SSD
Network	Mellanox MT27520 56GbE
**Spark Configuration**	
#nodes	1 master, 2 workers
#executors	10 executors
#executorcores	50 cores

**Table 3:** Summary of caching on 14 workloads.

Workload	Input Size (GB) (10x)	Sufficient Cache Memory (GB)		Reduced Cache Memory (GB)		
		Provided Cache Size (GB)	Max Required Cache Size (GB)	Provided Cache Size (GB)	Max Required Cache Size (GB)	
			CCA * & CAO ${}^{†}$		CCA *	CAO ${}^{†}$
Alternating Least Squares (ALS)	3.00	117.36	25.96	12.98	11.85	25.96
K-means clustering (KM)	18.70		40.22	20.11	19.74	40.22
Support Vector Machine (SVM)	18.63		40.97	20.49	11.17	40.97
Logistic Regression (LR)	22.4		49.20	24.60	22.35	49.20
Bayesian Classification (Bayes)	21.02		43.79	21.89	21.34	43.79
Linear Regression (LinR)	44.8		80.20	40.10	40.10	80.20
Random Forest (RF)	14.80		34.72	17.36	14.90	34.72
Latent Dirichlet Allocation (LDA)	2.10		4.97	2.49	2.46	4.97
Principal Components Analysis (PCA)	0.28		0.67	0.33	0.33	0.67
Gradient Boosting Trees (GBT)	0.30		2.44	1.22	1.11	2.54
Singular Value Decomposition (SVD)	5.00		5.00	2.50	0.00	5.00
PageRank (PR)	4.00		16.89	8.45	0.00	16.89
NWeight (NW)	0.70		3.02	1.51	1.38	3.02
TeraSort (TS)	40.00		40.00	20.00	0.00	40.00

* Cost-capacity-aware (proposed), ${}^{†}$ Cost-aware-only.

**Table 4:** Comparison between CCA and related studies.

	Execution	Operator	Memory	Optimizing
	Flow-Aware	Cost-Aware	Capacity-Aware	Execution Flow
MDF	O	X	X	O
S-CACHE	O	O	X	O
LRC	O	X	O	X
MRD	O	X	O	X
Neutrino	O	X	O	X
MemTune	X	X	O	X
LCS	O	O	O	X
CCA(proposed)	O	O	O	O

## Data Availability

Data sets used in this paper are given in the references cited.
